# Risk factors for perioperative stroke, myocardial infarction, and death in patients undergoing carotid endarterectomy under local anesthesia: a systematic review and meta-analysis

**DOI:** 10.3389/fsurg.2025.1677867

**Published:** 2026-01-22

**Authors:** Alessandra Ciccozzi, Diletta Riccio, Alba Piroli, Ida Marsili, Roberta Mariani, Federico Murgia, Chiara Angeletti, Paolo Matteo Angeletti, Daniele Tienforti, Franco Marinangeli, Arcangelo Barbonetti

**Affiliations:** 1 Department of Life, Health and Environmental Sciences-University of L’Aquila, L’Aquila, Italy; 2 Department of Anesthesia and Intensive Care San Salvatore University Hospital of L’Aquila, L'Aquila, Italy; 3 Department of Anesthesia, Intensive Care and Pain Therapy, Hospital Giuseppe Mazzini, Teramo, Italy; 4 Cardiovascular Department, Unit of Cardiac Anesthesia of the IRCCS Humanitas Research Hospital, Milano, Italy; 5 Andrology Unit, Department of Life, Health and Environmental Sciences-University of L’Aquila, L’Aquila, Italy

**Keywords:** carotid endarterectomy, death, local anesthesia, myocardial infarction, risk stratification, stroke

## Abstract

**Background:**

Patients with vascular disease undergoing surgery face increased perioperative risks, and those scheduled for carotid endarterectomy (CEA) represent a particularly vulnerable subgroup. This study aimed to (1) estimate the prevalence and identify predictors of adverse postoperative outcomes in patients undergoing carotid endarterectomy (CEA) under local/regional anesthesia (LA), and (2) compare these outcomes with those of general anesthesia (GA) where comparative data were available.

**Methods:**

Following PRISMA and MOOSE guidelines, PubMed, Scopus, and Web of Science were systematically searched for English-language studies published up to January 2025. Pooled prevalence estimates were obtained using random-effects models. Meta-regression explored associations of demographic and clinical variables with postoperative outcomes. In addition, pairwise random-effects meta-analyses were performed for studies reporting separate outcomes for LA and GA. Effect sizes were expressed as odds ratios (OR) with 95% confidence intervals (CIs), and heterogeneity was quantified using the I^2^ statistic.

**Results:**

Of 267 records identified, 14 studies met eligibility criteria, including 22,302 patients undergoing CEA under LA. The pooled prevalence was 1% for stroke (95% CI: 0.01–0.02) and 0.01% for both myocardial infarction and death (95% CI: 0.00–0.01). Meta-regressions showed that male sex was significantly associated with postoperative stroke (*β* = 0.010, *p* = 0.0002), whereas older age predicted myocardial infarction (*β* = 0.006, *p* = 0.03). No significant predictors of mortality were identified. In the comparative analysis, LA was associated with a 52% lower risk of myocardial infarction and a 30% lower risk of death compared with GA, while no significant difference emerged for postoperative stroke.

**Conclusion:**

CEA performed under regional anesthesia is associated with low rates of adverse postoperative events, with male sex and older age emerging as relevant predictors for stroke and myocardial infarction, respectively. Comparative evidence suggests potential advantages of LA over GA in reducing myocardial infarction and mortality, while stroke risk appears similar between anesthetic modalities.

**Systematic Review Registration:**

https://www.crd.york.ac.uk/PROSPERO/, PROSPERO CRD420251066377.

## Introduction

Carotid atherosclerosis, specifically in symptomatic individuals with severe stenosis (≥70%), is a well-established indication for surgical intervention. In such cases, carotid endarterectomy (CEA) may reduce the risk of stroke by 79%–80% (1). However, the clinical benefits of CEA in patients with moderate or asymptomatic stenosis remain debatable. Although CEA tends to yield favorable outcomes in individuals with low perioperative risk, many patients with vascular diseases are inherently at high risk and frequently present with significant cardiovascular and cerebrovascular comorbidities. Notably, major adverse cardiac events and major cerebrovascular events are not uncommon in this population (2). The impact of the anesthetic technique (local vs. general) on postoperative outcomes following CEA remains inconclusive. Although some studies have highlighted the theoretical advantages of regional anesthesia, definitive evidence supporting its superiority is lacking (3).

Age is another critical demographic factor that often compounds the perioperative risk owing to its association with frailty (4). Accordingly, international guidelines recommend the use of clinical risk assessment tools to identify patients at increased risk of postoperative complications. Among these tools, the Vascular Study Group of New England Cardiac Risk Index (5) has been proposed to improve perioperative risk stratification in vascular surgery. Integrating objective biomarkers, such as pro-BNP and hemoglobin, with clinical variables, including age, functional status, and American Society of Anesthesiologists classification, may improve the predictive accuracy of traditional risk models. A recent study demonstrated that such multifactorial approaches improve the identification of high-risk patients undergoing CEA. Nonetheless, substantial uncertainty remains regarding the optimal anesthetic strategy for CEA, particularly in high-risk patients, and its impact on both short- and long-term outcomes (6). Therefore, according to the PICO model (Population, Intervention, Comparator, Outcome, Supplementary Table S1) (7), this systematic review and meta-analysis was designed with a dual objective: (1) to estimate the prevalence of adverse outcomes and identify their predictors in high-risk patients undergoing carotid endarterectomy (P) under regional anesthesia (I), and (2) to perform a pairwise comparative meta-analysis of studies reporting separate outcomes for regional vs. general anesthesia (C), in order to evaluate the potential impact of anesthetic modality on postoperative prognosis (O).

## Methods

This meta-analysis adheres to the Cochrane Collaboration methodology, Preferred Reporting Items for Systematic Reviews and Meta-Analyses (PRISMA) statement (8), and Meta-analysis of Observational Studies in Epidemiology (MOOSE) guidelines (9). The PRISMA checklist is presented in Supplementary Table S2. This meta-analysis was registered in PROSPERO database (https://www.crd.york.ac.uk/PROSPERO/. ID: CRD420251066377).

### Literature search strategy

PubMed, Scopus, and Web of Science were systematically searched to identify all relevant English-language studies published until January 2025 using the following terms: state “carotid endarterectomy,” “local anesthesia,” “risk factors,” “stroke,” “myocardial infarction,” and “death.” Boolean operators (AND/OR) were used to combine key terms. If relevance could not be determined from the abstract, the full text was retrieved for assessment. Eligible studies were identified by two independent authors (D.R. and A.C.), and disagreements were resolved by a third researcher (A.B.). References cited in all the articles were manually searched to identify additional studies.

### Eligibility criteria

Studies were eligible for inclusion if they reported postoperative outcomes—stroke, myocardial infarction, and/or death—in patients undergoing carotid endarterectomy under local/regional anesthesia (CEA-LA), providing either the prevalence of these events or sufficient data to calculate it. Observational studies (case-control, cross-sectional, prospective cohorts, and case series) as well as intervention studies were screened for eligibility. For case-control studies, only outcome data pertaining to patients treated with LA were extracted for the prevalence analysis.

In addition, studies that reported separate outcome data for LA and general anesthesia (GA) were included in a complementary comparative analysis of the two anesthetic modalities. Included studies enrolled symptomatic, asymptomatic, or mixed populations. Because most primary studies did not stratify outcomes separately by symptom status, all eligible patients with stenosis >70% were included, and symptomatology was recorded as a descriptive variable when available.

Duplicates were rigorously checked and removed, and when multiple publications analyzed overlapping populations, only the study with the largest sample size was retained. Reviews, meta-analyses, and studies not reporting the outcomes of interest were excluded. Two independent reviewers (D.R. and A.C.) assessed full-text articles for eligibility, and disagreements were resolved by consensus with a third reviewer (A.B.).

### Data extraction

Two independent reviewers (D.R. and A.C.) extracted data from all eligible studies, including first author, year of publication, study design, demographic characteristics (sex distribution and mean age), total number of patients undergoing CEA, and the number of events for each postoperative outcome. When available, we also collected information on the proportion of patients with contralateral carotid artery stenosis >70% who did not undergo surgery, and the presence of preoperative cortical or ocular symptoms among symptomatic individuals. When reported, we also collected information on the type of regional anesthesia (superficial, deep, or combined cervical plexus block); however, most studies did not stratify outcomes by block type.

For studies providing separate outcome data for procedures performed under local/regional vs. general anesthesia, both sets of data were extracted to enable the comparative analysis.

Summary statistics not explicitly reported in the original articles were derived whenever possible (10). In cases of missing, incomplete, or inconsistent information, study authors were contacted to obtain clarification. All extracted data are presented in Table 1.

**Table 1:** Characteristics of the included studies.

Study	Design	Stroke (events)	AMI (events)	Death (events)	Mean age (years)	Type of regional anesthesia	Males (n/tot., %)	CLAS >70%	Symptomatic stenosis (%)
Orlicky et al. 2019	PRCS	2	0	0	68	CPB+LI	67/105 (63.8)	NR	60%
McCarthy et al. 2002	PRCS	0	1	1	72	SCB+LI	25/34 (73.5)	8	76%
Rocha-neves et al. 2021^a^	ROCS	14	NA	NA	70	SCB or DCB	131/164 (79.8)	164	40.5%
Kline et al. 2023	ROCS	57	26	12	71	NR	2,575/4,152 (62.0)	NR	NR
Mazzaccaro et al. 2019	ROCS	1	5	1	84	NR	102/178 (57.3)	NR	21–24%
Pasin et al. 2015^a^	ROCS	23	15	3	72	SCB and/or DCB	1,597/2,439 (65.5)	NA	27%
Knappich et al. 2019	Analysis of data from 5 RCTs	51	4	5	70	NR	951/1,332 (71.4)	111	100%
Chou et al. 2016	ROCS	85	25	37	72	NR	3,593/5,907 (60.8)	NR	38–40%
Dakour Aridi et al. 2018	ROCS	50	16	13	72	NR	4,179/6,684 (62.5)	NR	27–31%
Mendonça et al. 2014	POS	2	3	1	71	SCB and DCB	77/117 (65.8)	NR	55%
Birchley et al. 2010	POS	1	2	2	72	SCB and DCB	66/100 (66)	13	15%
Markovic et al. 2012	Mixed (POS and ROCS)	3	3	3	66	SCB and DCB	494/773 (63.9)	NR	58–63%
Sternbach et al. 2002^a^	ROCS	4	1	2	72	SCB	136/226 (60.2)	NR	37–45%
McCarthy et al. 2001	POS	2	1	2	71	SCB	71/91 (78.0)	37	83–95%

AMI, acute myocardial infarction; CLAS>70%: number of patients with a contralateral artery stenosis of more than 70%; CPB, cervical plexus block; DCB, deep cervical block; LI, local infiltration; NR, not reported; POS, prospective observational study; PRCS, prospective randomized controlled study; RCT, randomized controlled trial; ROCS, retrospective observational cohort study; SCB, superficial cervical block.

a Study conducted in a high-volume center (performing more than 100 carotid endarterectomy interventions per year).

### Quality assessment

The Effective Public Health Practice Project Quality Assessment Tool (11) was used to assess the methodological quality of the included studies. This assessment tool, designed for intervention studies such as randomized controlled trials and case-control studies, has been validated for use in systematic reviews and evaluates six domains: selection bias, study design, confounding factors, study blindness, data collection method, and loss to follow-up. The quality of each domain is rated as strong, moderate, or weak. Overall study quality is classified as “strong,” if no domains are rated as weak, “moderate,” if only one domain is rated as weak, and “weak,” if two or more domains are rated as weak. The assessments were performed independently by two reviewers (D.R. and D.T.), and any discrepancies were resolved by the third reviewer (A.C.).

### Statistical analysis

The pooled prevalence of adverse outcomes of interest (stroke, myocardial infarction, and death) was estimated using a random effects model, which assumes that the included studies have varying effect sizes, providing a conservative estimate of the overall effect. The 95% confidence intervals (CIs) of the prevalence reported in individual studies were estimated from the proportion of event cases and sample size using the binomial Clopper–Pearson exact method. After ascertaining the non-normal distribution of the original datasets (using the Shapiro–Wilk test), the Freeman–Tukey double arcsine transformation was applied to the primary study data to approximate normality. The final pooled results and 95% CIs were back-transformed and expressed as percentages for easier interpretation. The inverse variance method was used to weigh each study in the pooled estimates. Among the included studies, some provided separate outcome data for patients undergoing CEA under local/regional anesthesia (LA) vs. general anesthesia (GA). For these studies, we performed additional pairwise random-effects meta-analyses comparing GEA-LA and GA for each of the three primary endpoints (stroke, myocardial infarction, and death), expressing effect estimates as odds ratios (OR) with 95% CI. These analyses were conducted using the same statistical framework adopted for the prevalence meta-analysis, ensuring methodological consistency. Statistical heterogeneity between the results of different studies was analyzed using the Cochran's Chi-square (Cochran's Q) and the I^2^ tests. An I^2^ > 50% and/or *p* < 0.05 indicated substantial heterogeneity (12). Covariates that could affect the estimates, such as mean age, sex, and the proportion of patients who experienced preoperative cortical or ocular symptoms, were included in the linear meta-regression models. Data were analyzed and graphed using the packages “metafor” and “ggplot2” of the R statistical software (version 4.4.1, 2024; The R Foundation for Statistical Computing, Vienna, Austria) and the Review Manager software of the Cochrane Library (version5.3; The Nordic Cochrane Centre, The Cochrane Collaboration, Copenhagen, Denmark).

## Results

**Figure 1:**
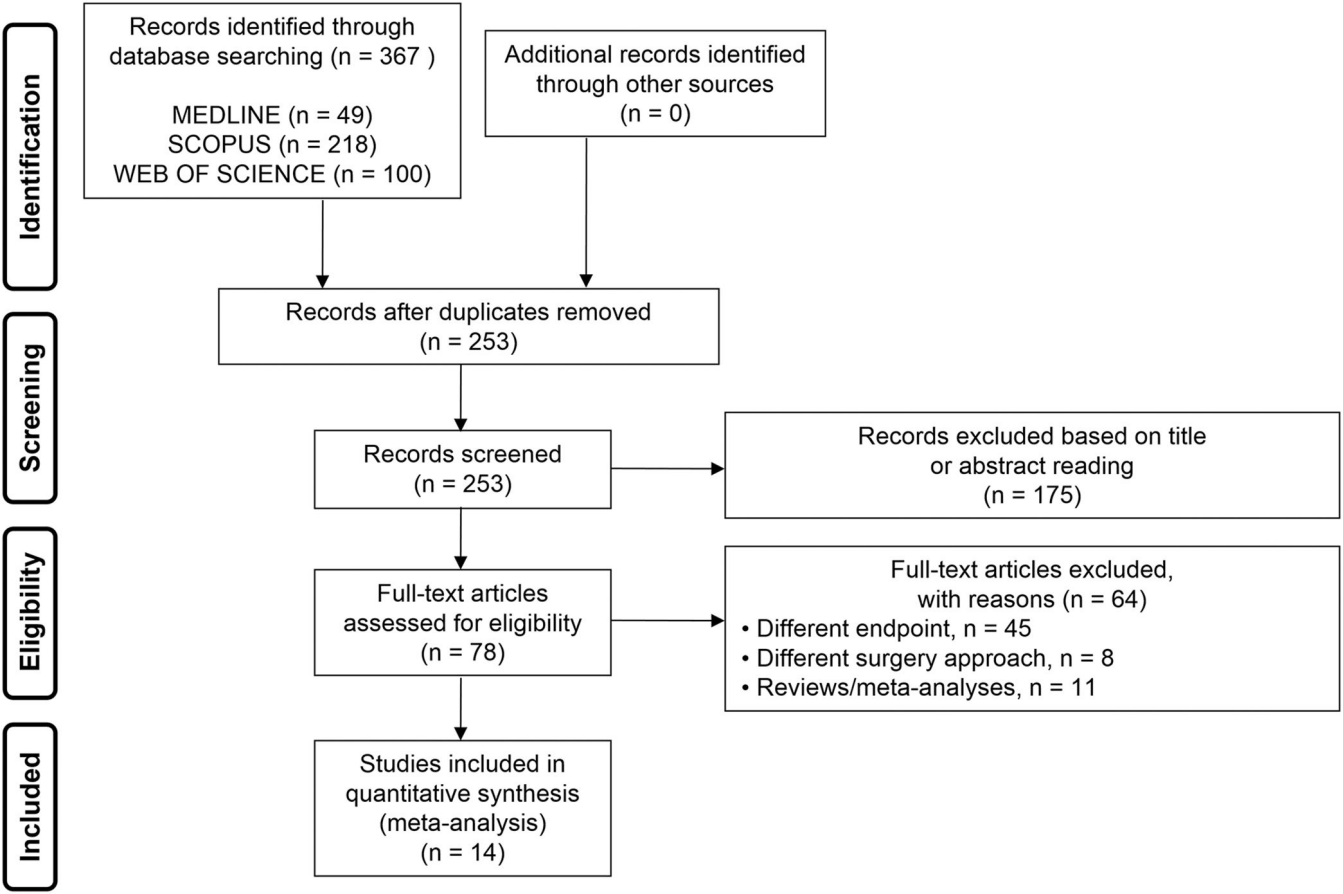
Flow diagram showing an overview of the study selection process.

A total of 367 studies were initially identified, of which 253 remained after removing the duplicates. Based on titles and abstracts, 175 studies were excluded as irrelevant. Of the remaining 78 studies, 14 met the inclusion criteria (Figure 1) (13–26).

These 14 included studies collectively provided information on 22,302 patients undergoing CEA-LA. Main characteristics of the included studies are presented in Table 1.

### Quality assessment of the studies

The methodological quality of the included studies, according to the Effective Public Health Practice Project Quality Assessment Tool, is presented in Table 2. Overall, all studies were rated as moderate in global quality. A consistent pattern emerged across domains: the blinding component was uniformly judged as weak, largely due to the inherent challenges in masking participants and outcome assessors in the study designs considered. Conversely, the data collection methods were consistently rated as strong, reflecting the use of validated instruments, standardized protocols, and clearly reported measurement procedures. Other domains—such as selection bias, study design, confounders, and withdrawals/dropouts—showed variable ratings across studies but did not substantially alter the overall moderate classification. Taken together, the body of evidence can be considered methodologically sound, with strengths in data collection balanced by predictable limitations related to blinding.

**Table 2:** Quality assessment of the included studies.

Study	Selection bias	Study design	Confounders	Blinding	Data collection methods	Withdrawals and drop-outs	Global rating
Orlicky et al. 2019	Moderate	Strong	Moderate	Weak	Strong	Strong	Moderate
McCarthy et al. 2002	Moderate	Strong	Strong	Weak	Strong	Strong	Moderate
Rocha-Neves et al. 2021	Moderate	Strong	Strong	Weak	Strong	Strong	Moderate
Kline et al. 2023	Moderate	Moderate	Strong	Weak	Strong	Moderate	Moderate
Mazzaccaro et al. 2019	Moderate	Moderate	Moderate	Weak	Strong	Moderate	Moderate
Pasin et al. 2015	Moderate	Moderate	Strong	Weak	Strong	Moderate	Moderate
Knappich et al. 2019	Moderate	Strong	Strong	Weak	Strong	Moderate	Moderate
Chou et al. 2016	Moderate	Moderate	Moderate	Weak	Strong	Moderate	Moderate
Dakour Aridi et al. 2018	Moderate	Moderate	Strong	Weak	Strong	Moderate	Moderate
Mendonça et al. 2014	Moderate	Moderate	Moderate	Weak	Strong	Moderate	Moderate
Birchley et al. 2010	Moderate	Moderate	Moderate	Weak	Strong	Moderate	Moderate
Markovic et al. 2012	Moderate	Moderate	Strong	Weak	Strong	Moderate	Moderate
Sternbach et al. 2002	Moderate	Moderate	Strong	Weak	Strong	Moderate	Moderate
McCarthy et al. 2001	Moderate	Moderate	Moderate	Weak	Strong	Moderate	Moderate

### Synthesis of results of prevalence meta-analysis and meta-regression analysis

#### Stroke

**Figure 2:**
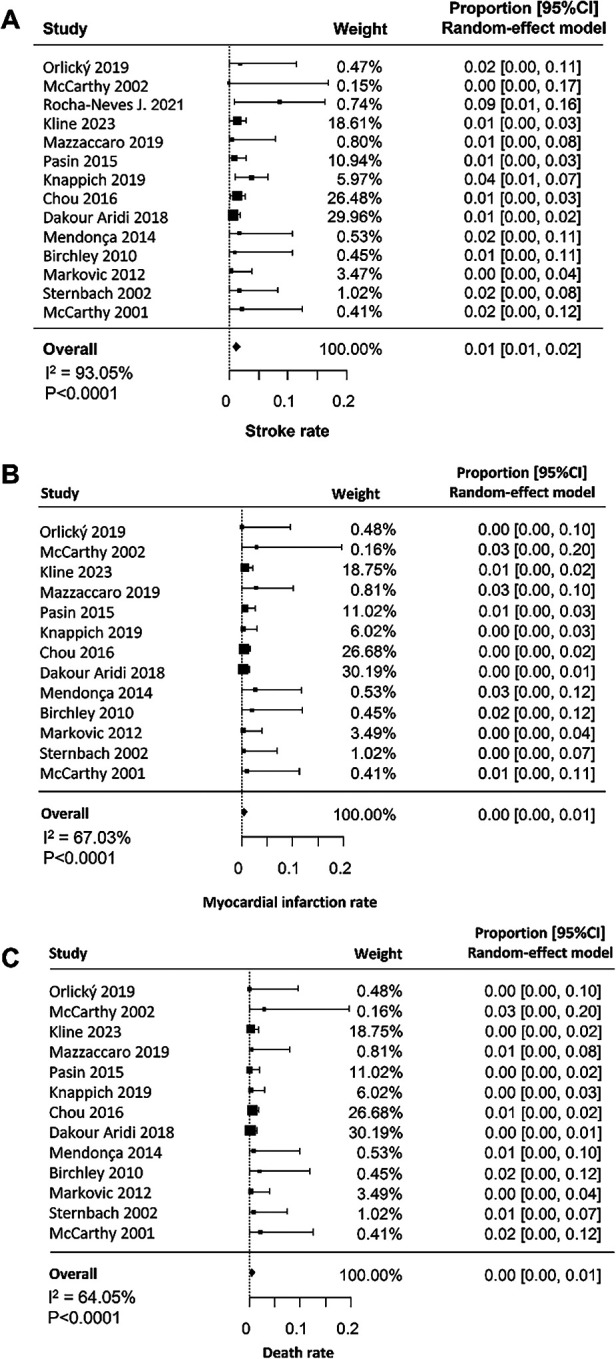
Forest plots depicting the pooled prevalence estimate for stroke **(A)**, myocardial infarction **(B)** and death rate **(C)** in patients undergoing carotid endarterectomy. Diamonds indicate the overall summary estimates, and the width of the diamonds represents the 95% confidence interval (CI); boxes indicate the weight of individual studies in the pooled result.

**Figure 3:**
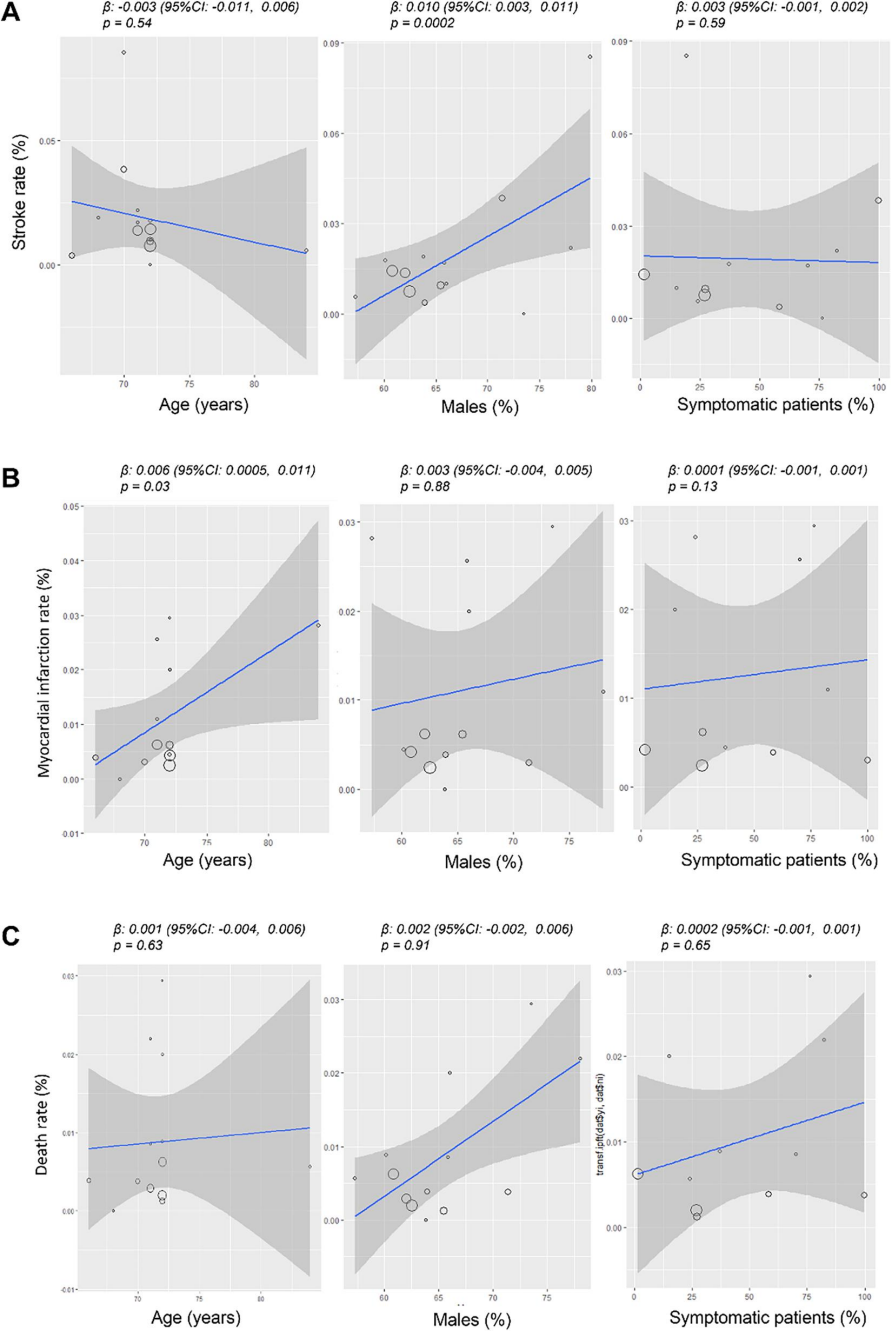
Meta-regression bubble plots: rate of stroke **(A)**, acute myocardial infarction **(B)** and death **(C)** in patients undergoing carotid endarterectomy as a function of the mean age, percentage of males and symptomatic patients in the study population. The predicted effects (solid line) with corresponding confidence intervals (gray range) are also shown. CI, confidence interval.

The overall rate of stroke was 1% (95% CI: 0.01–0.02; Figure 2A), with high and significant heterogeneity across studies (I^2^ = 93%, *p* < 0.0001). In the meta-regression analysis, post-CEA was not significantly associated with age (*β* = ˗0.003, 95% CI: ˗0.011–0.006, *p* = 0.54) or with the proportion of pre-CEA symptomatic patients (*β* = 0.003, 95% CI: ˗0.001–0.002, *p* = 0.59). In contrast, the stroke rate increased significantly with a higher percentage of male patients in the study populations (*β* = 0.010, 95% CI: 0.003–0.011, *p* = 0.0002) (Figure 3A).

#### Acute myocardial infarction

The overall rate of acute myocardial infarction was 0.0% (95% CI: 0.00–0.01; Figure 2B), with significant heterogeneity across studies (I^2^ = 67%, *p* < 0.0001). In the meta-regression analysis, post-CEA acute myocardial infarction was not significantly associated with male sex (*β* = 0.003, 95% CI: ˗0.004–0.005, *p* = 0.88) or with the proportion of pre-CEA symptomatic patients (*β* = 0.0001, 95% CI: ˗0.001–0.001, *p* = 0.23). In contrast, the rate of myocardial infarction increased significantly with advancing age in the study populations (*β* = 0.006, 95% CI: 0.0005–0.011, *p* = 0.03) (Figure 3B).

#### Mortality

The overall death rate was 0.0% (95% CI: 0.00–0.01; Figure 2C), with significant heterogeneity across studies (I^2^ = 64%, *p* < 0.0001). In the meta-regression analysis, post-CEA mortality was not significantly associated with age (*β* = 0.001, 95% CI: ˗0.004–0.006, *p* = 0.63), the proportion of male patients in the study populations (*β* = 0.002, 95% CI: ˗0.002–0.006, *p* = 0.91), or the proportion of pre-CEA symptomatic patients (*β* = 0.0002, 95% CI: ˗0.001–0.001, *p* = 0.65) (Figure 3C).

### Pairwise comparative meta-analysis

Some of the included studies reported separate postoperative outcomes for CEA performed under local/regional anesthesia (LA) and general anesthesia (GA). These studies were pooled in a pairwise random-effects meta-analysis to compare the two anesthetic modalities.

**Figure 4:**
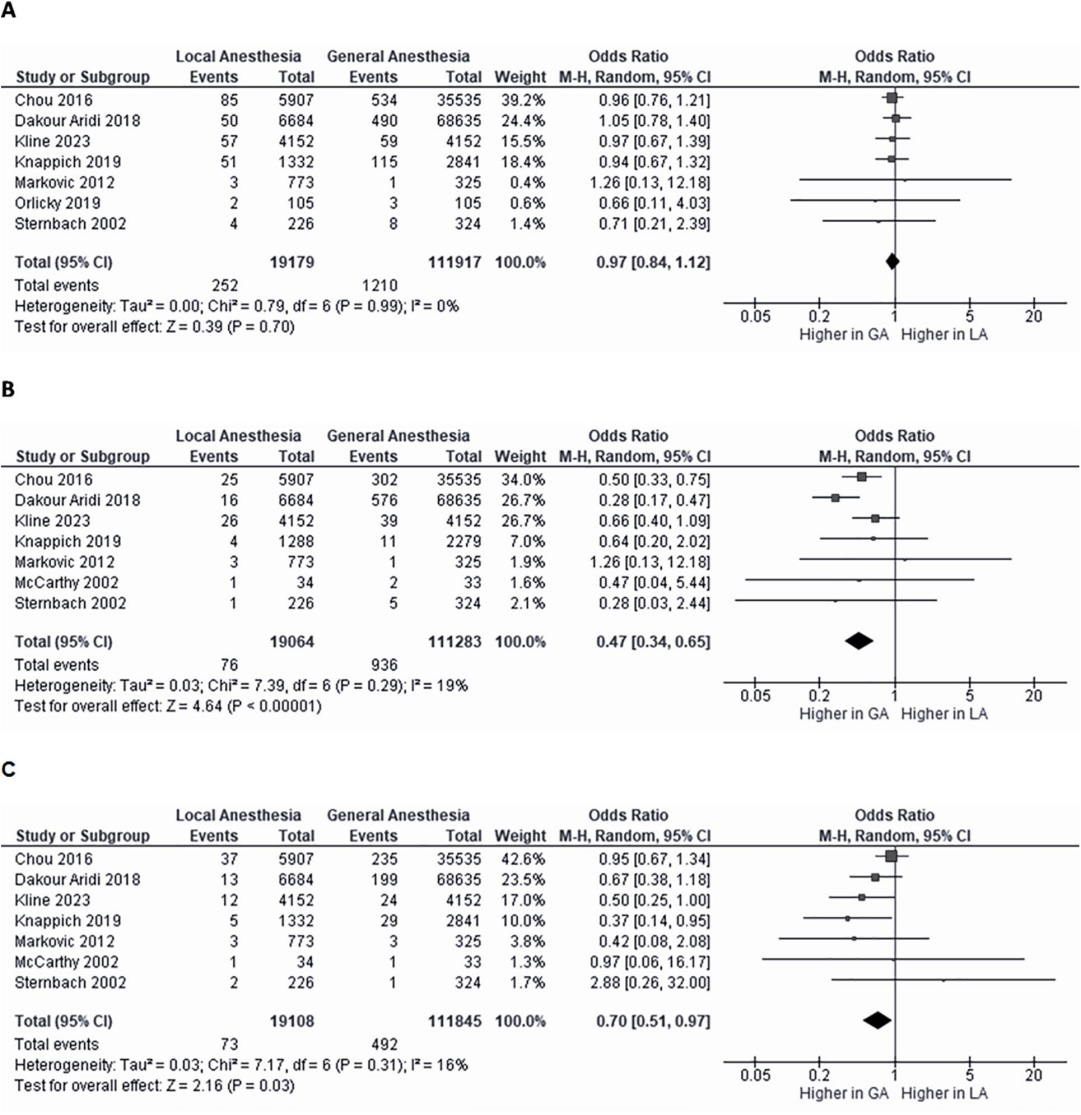
Pairwise comparative meta-analysis of local/regional anesthesia versus general anesthesia in carotid endarterectomy. Forest plots comparing postoperative stroke **(A)**, myocardial infarction **(B)** and death rate **(C)** between local/regional anesthesia (LA) and general anesthesia (GA). Effect sizes are expressed as odds ratios (OR) with 95% confidence intervals, calculated using a random-effects model. Diamonds indicate the overall summary estimates, and the width of the diamonds represents the 95% confidence interval (CI); boxes indicate the weight of individual studies in the pooled result. df, degrees of freedom; M-H, Mantel-Haenszel.

LA and GA showed no significant difference in postoperative stroke incidence (Figure 4A).

In contrast, LA was associated with a 52% lower risk of myocardial infarction (Figure 4B) and a 30% lower risk of death compared with GA (Figure 4C).

## Discussion

To the best of our knowledge, this is one of the few prevalence meta-analyses evaluating the incidence and predictors of adverse cardiovascular and cerebrovascular events in patients undergoing CEA-LA. Meta-regression analyses identified male sex as significantly associated with a higher incidence of postoperative stroke, whereas increasing age was correlated with an elevated risk of myocardial infarction. Interestingly, none of the three evaluated variables (age, sex, or preoperative neurological symptoms) were significantly associated with postoperative mortality. This contrasts with previous studies, including that of Leung (27), which reported a linear increase in all three outcomes with advancing age in symptomatic patients. Our results indicate a more nuanced interplay between individual risk factors and postoperative outcomes.

These findings are also consistent with earlier evidence showing variability in perioperative outcomes after CEA, as reported by Pistolese et al. (28), who highlighted the influence of patient-specific characteristics on postoperative neurological and cardiac complications. The substantial heterogeneity observed in the prevalence meta-analysis, particularly in stroke incidence, may be partially attributed to differences in sample size and methodological quality across the included studies. For instance, studies by Dakour Aridi (2018) (21) and Chou (2016) (20) had a greater influence on pooled estimates because of their larger cohorts and narrower confidence intervals, which contributed to greater statistical precision. Conversely, smaller studies such as those by McCarthy (2002) (14) and Mendonca (2014) (22), despite reporting higher stroke rates, had limited influence owing to their lower weighting and wider variability. The heterogeneity observed across studies may partly reflect also differences in study design, as both prospective and retrospective cohorts were included (Table 1).

In addition to the prevalence meta-analysis restricted to CEA under regional anesthesia, our study incorporated a complementary pairwise comparison between LA and GA in those studies reporting separate outcomes. These analyses revealed that LA was associated with significantly lower rates of postoperative myocardial infarction (−52%) and mortality (−30%), whereas no significant advantage was observed for stroke. This pattern is consistent with the physiologic rationale that LA may reduce perioperative hemodynamic stress and myocardial oxygen demand and aligns with previous evidence including the GALA Trial (3), where MI and cardiac complications tended to favor LA despite no difference in stroke incidence. The lack of stroke difference reinforces the notion that stroke risk during CEA is primarily driven by surgical and plaque-related factors, rather than anesthetic modality.

An important limitation of our analysis is the limited number of variables evaluated. Many primary studies lacked data on potentially influential clinical factors such as comorbidities, smoking status, and thrombophilic conditions, thereby precluding more detailed subgroup or sensitivity analyses. The absence of such information limited our ability to explore deeper interactions and develop more comprehensive predictive models. Moreover, the volume of the surgical centers (high- vs. low-volume) was inconsistently reported (Table 1) and could not be incorporated into the analysis, although it is a known factor influencing perioperative outcomes.

The overall low incidence of myocardial infarction and mortality is encouraging, suggesting that, with appropriate patient selection, CEA-LA can be performed with acceptable safety. However, the observed associations between stroke and male sex and between myocardial infarction and advancing age highlight the need for careful preoperative evaluation.

Identifying demographic variables that significantly influence outcomes may support individualized patient selection and guide intraoperative decision-making. In vascular surgery settings, often involving patients with multiple comorbidities and elevated cardiovascular risk, such insights are valuable for minimizing adverse outcomes.

In conclusion, this meta-analysis suggests that CEA-LA is associated with a low but clinically significant risk of adverse outcomes, particularly in specific subpopulations. Male sex was identified as a predictor of postoperative stroke, whereas older age was associated with an increased risk of myocardial infarction. Although overall mortality was low and not significantly influenced by the variables assessed, inter-study heterogeneity remains a limitation. The potential advantages of LA over GA in reducing myocardial infarction and mortality showed by comparative evidence has an important impact on the choice of anesthetic technique, local vs. general, in patients with cardiovascular comorbidity undergoing CEA. These results underscore the need for individualized perioperative planning and highlight the importance of long-term follow-up to ensure both the efficacy and safety of CEA-LA.

## Data Availability

The original contributions presented in the study are included in the article/Supplementary Material, further inquiries can be directed to the corresponding author/s.
