# Supplementary material for: Risk factors for perioperative stroke, myocardial infarction, and death in patients undergoing carotid endarterectomy under local anesthesia: a systematic review and meta-analysis
Source: Front Surg. 2026 Jan 22;12:1677867. doi: 10.3389/fsurg.2025.1677867 (PMC12872913; doi:10.3389/fsurg.2025.1677867)
Supplement: Supplementary file 1 [file Table1.docx]

# Supplementary Table 1. PICO framework of the study

| **Component** | **Description** |
| --- | --- |
| Population (**P**) | High-risk adult patients undergoing carotid endarterectomy (CEA). |
| Intervention (**I**) | Carotid endarterectomy performed under regional/local anesthesia (LA). |
| Comparator (**C**) | General anesthesia (GA), applicable only to the subset of included studies reporting separate outcome data for both anesthetic techniques. |
| Outcomes (**O**) | (1) Prevalence of postoperative stroke, myocardial infarction, and death in CEA performed under LA; (2) Predictors of these outcomes within LA-treated patients; (3) Comparative postoperative risk of stroke, myocardial infarction, and death between LA and GA. |
| Study design | Systematic review with prevalence meta-analysis and additional pairwise comparative meta-analysis. |

Footnote: The Comparator (C) was included only for studies providing separate outcomes for patients undergoing carotid endarterectomy under regional versus general anesthesia.
